# Prevalence of Overnight Work (1 a.m. to 5 a.m.) Among United States Workers

**DOI:** 10.1002/ajim.70027

**Published:** 2025-10-07

**Authors:** Imelda S. Wong, Toni Alterman, Beverly M. Hittle, Raquel Velazquez‐Kronen, I‐Chen Chen

**Affiliations:** ^1^ Office of the Provincial Health Officer, Ministry of Health Government of British Columbia Victoria British Columbia Canada; ^2^ Colorado School of Public Health, Centers for Health, Work & Environment CU Anshutz Aurora Colorado USA; ^3^ Division of Field Studies and Engineering National Institute for Occupational Safety and Health Cincinnati Ohio USA; ^4^ College of Nursing University of Cincinnati Cincinnati Ohio USA; ^5^ State of Hawaii Department of Human Services Honolulu Hawaii USA

**Keywords:** industry, National Health Interview Survey, night shift, nonstandard work schedule, occupation, overnight work, prevalence, shift work, US workers, work hours

## Abstract

**Background:**

Many factors have resulted in the normalization of nonstandard work schedules in recent decades, including globalization requiring working across time zones and growing demands for goods and services. This paper provides national estimates of overnight work in the USA.

**Methods:**

We used cross‐sectional data from the 2015 National Health Interview Survey (*n* = 19,386 US employed adults ≥ 18 years). This survey contained a unique definition of overnight work (i.e., between 1:00 a.m. and 5:00 a.m.), based on the window of circadian low. Weighted prevalence rates were provided across categories of sociodemographic characteristics, health status, health behaviors, and occupational factors.

**Results:**

We estimated more than 21 million US employed adults experienced overnight work (14.2%). Higher prevalence was found among men (17.8%), non‐Hispanic Black adults (17.2%), non‐US born adults (11.2%), those with some college (15.9%) or a high school (16.7%) education, or living in the Midwest region (15.8%). Compared to those sleeping 7–9 h (10.5%), higher percentages of adults working overnight slept < 7 h (21.4%) and > 9 h (17.0%). Increasing prevalence was observed with increasing weekly work hours (*p* < 0.0001). Higher prevalence was reported among multiple job holders (19.5%). Industries and occupations with the greatest percentage of overnight workers were Transportation, Warehousing and Utilities (29.3%), and Protective Services (47.4%).

**Conclusion:**

Our estimates of overnight work in 2015 are almost five times higher than estimates from 2004. Given that overnight work has been associated with adverse safety and health outcomes, additional policies and programs are needed to protect this growing population of workers.

## 1 Introduction

Work scheduled outside of regular daytime hours, such as overnight shifts, is associated with a wide range of negative health and safety outcomes [1, 2, 3, 4, 5]. It is hypothesized that working during normal sleeping hours and sleeping during normal waking hours can lead to desynchronization of circadian rhythms [1, 6, 7]. Circadian rhythms act as internal clocks in our bodies which drive virtually all physiologic and behavioral processes [6, 8]. Disruption of regular circadian functioning may be the primary biological pathway linking overnight shifts with adverse outcomes [8]. In the short term, this could lead to impaired sleep and cognition with further increased risks for work injury [9, 10, 11]. Prolonged exposure to overnight work has been associated with increased risk of chronic health conditions, such as cardiovascular disease, diabetes, and cancer [1, 4, 5, 6, 12, 13].

In addition to circadian disruption, other mechanisms or pathways may link shift work with increased risks among overnight shift workers. For example, nonstandard schedules, such as night shifts, have been described as “asynchronous with the majority of society” and may lead to feelings of time scarcity and social and psychological distress [14]. Night shift workers may have difficulty finding time to attend social events in the evening and may forego sleep to manage family responsibilities, school, or other employment during the day [15, 16]. Schedules involving “chronobiological and social disruption (e.g., night shifts),” are also linked to unhealthy behaviors, such as frequent smoking, heavy alcohol consumption and sedentary behaviors [14, 17].

The prevalence of overnight work in the USA has been explored in prior studies using the Current Population Surveys (CPS) [18, 19, 20, 21]. Presser employed the 1991 and 1997 CPS questions regarding start and end times of the respondent's main job in the week prior, to define “fixed night” shifts as “at least half the hours worked most days last week falling between midnight and 8 a.m.” [18, 19, 22, 23]. Beers and McNenamin used the 1997 and 2004 CPS data, respectively, in which respondents were asked to self‐select the type of work schedule which best described their work at their main job [20, 21, 23, 24]. “Regular night shift” in the CPS was defined as “anytime around 9 p.m. to 8 a.m.” However, over the past few decades, nonstandard work schedules have become more common, due in part from globalization requiring working across time zones, advances in information communications technologies, and growing demands for goods and services [14, 25, 26]. As such, it is unclear if prior prevalence estimates reflect the current US workforce.

As demonstrated by existing literature, definitions of shift work in previous epidemiologic studies have varied widely, leading to calls for more precise measures to improve exposure assessment and risk estimates [27]. Prior studies have used broad categories of shift work (e.g., day, evening, night, rotating), which may result in exposure misclassification [27, 28]. The International Agency for Research on Cancer (IARC) has recommended several “domains” to improve exposure assessment of night shifts, including the identification of work between midnight and 5 a.m., which has the most substantial effects on “circadian phase shifts and sleep perturbation” [29]. Similarly, the “window of circadian low” has been defined as the hours between 2:00 a.m. and 6:00 a.m., and represents the period of clock time where individuals exhibit peak fatigue and greatest performance decrements [30, 31, 32].

This paper builds upon prior surveillance studies of overnight work to provide updated national prevalence estimates for the USA. Our objective was to describe the prevalence of overnight work across sociodemographic characteristics, health status, health behaviors, and occupational factors.

## 2 Methods

### 2.1 Data Source

We used publicly available data from the 2015 National Health Interview Survey (NHIS), a nationally representative cross‐sectional survey of health among noninstitutionalized civilians residing in the United States [33, 34]. While the 2015 survey may not reflect the most current workforce characteristics (e.g., rise in overnight work with the increased globalization of work and the gig economy) [35, 36], it includes a NIOSH‐sponsored occupational health supplement with a precise definition of overnight work, based on clock‐time and circadian influences, that has not been repeated in subsequent years. The most recent NHIS that included a question about work hours was in 2021, which asked about “usual hours of work.” However, respondents self‐selected into five broad shift work categories which did not include any clock‐time definitions (e.g., day shift, evening shift, night shift, rotating shift, some other shift).

The NHIS is an annual survey conducted by the National Center for Health Statistics, Centers for Disease Control and Prevention, using a multistage, clustered, and stratified area probability design that permits representative sampling of US households and other noninstitutional dwellings. Trained representatives from the US Census Bureau conducted in‐person computer‐assisted interviews (with some telephone follow‐up). Certain populations, such as Black, Hispanic, Asian populations, and those aged 65 or older, were oversampled. All respondents provided verbal consent prior to participating [33, 37]. The NHIS is approved by the Ethics Review Board of the National Center for Health Statistics and the U.S. Office of Management and Budget [33].

In 2015, the NHIS obtained data from 41,293 households representing 103,789 individuals [33]. The number of sample adults surveyed was 33,672, with a response rate of 55.2% [33]. We restricted our study population to respondents aged 18 years and older who were defined as “currently employed” in the NHIS. This includes those who, in the week prior to being interviewed, had worked either for pay at a job or business, with a job or business but not at work, or working without pay at a family‐owned job or business [33].

### 2.2 Study Definitions

#### 2.2.1 Overnight Work

Currently employed adult participants were asked the following question pertaining to overnight work: “During the past 30 days, did you work any amount of time between 1:00 a.m. and 5:00 a.m.?” This question was included in an occupational health supplement sponsored by the National Institute for Occupational Safety and Health and was developed in consultation with sleep and shift work experts. This definition is similar to others, such as the “window of circadian low,” and follows recommendations from the IARC committee who first identified shift work as a probable human carcinogen [29, 30, 31, 32]. We further described the national prevalence of overnight work by sociodemographic characteristics, health status, health behaviors, and occupational factors. Definitions for variables used in this study and associated NHIS survey questions are included in Supplement S1A.

#### 2.2.2 Sociodemographic Variables

Respondents' sociodemographic characteristics included self‐reported age, sex, race and ethnicity, region of residence, highest level of education attainment, marital status, and presence of minor children in the family. Race and ethnicity categories reflect the questions used in the 2015 survey and OMB 1997 standards for reporting. We categorized nativity as “US born” or “non‐US born.”

#### 2.2.3 Health Status and Health Behaviors

Health status and health behaviors included self‐reported health, leisure‐time physical activity, sleep, smoking, and alcohol use. Self‐reported health was categorized as excellent/very good, good, and fair/poor. Psychometric studies recommend combining “excellent” and “very good” categories to achieve better linear fit with the general health evaluation concept, because the difference between these two categories is significantly smaller than between other categories [38, 39, 40]. Sleep was ascertained by the question “On average, how many hours of sleep do you get in a 24‐h period?”. We used “7–9 h” as the reference category, based on sleep duration recommendations for healthy adults to promote optimal health [41, 42].

The NHIS categorized leisure‐time physical activities as “vigorous” or “light or moderate” based on self‐reported usual weekly frequency and intensity. “Vigorous” activity was defined in the NHIS as activities lasting “at least 10 min that cause heavy sweating or large increases in breathing or heart rate.” “Light or moderate” activity was defined as those lasting “at least 10 min that cause only light sweating or a slight to moderate increase in breathing or heart rate” [33]. Smoking status was defined based on lifetime and current smoking behaviors. All adults were asked if they had smoked at least 100 cigarettes in their entire life. Those who said “yes” were asked a series of questions about the age at which they began smoking and their current smoking practices (every day, some days, not at all). The NHIS defines current smokers as those who have ever smoked 100 cigarettes and currently smoke every day or some days. Those who no longer smoked were categorized as former smokers [33]. We combined the five NHIS categories (“current every day,” “current some day,” “former,” “never,” “smoker, current unknown”) into three categories representing “never,” “former,” and “current” smoker. Assessment of participants' alcohol use was similarly ascertained from a series of questions about lifetime and current consumption and categorized as “infrequent,” “light,” “moderate,” and “heavy” [33, 34].

#### 2.2.4 Occupational Characteristics

Additional occupational characteristics included self‐reported weekly work hours, usual work schedule, years on the job, work arrangement, employer type, multiple jobs, paid sick leave, industry, and occupation. Weekly hours worked were assessed with the question “How many hours did you work last week at all jobs or businesses?” Categories of work hours were centered around the 40‐h workweek limit identified by the Fair Labor Standards Act (§207(a)), after which overtime pay is required for certain categories of workers [43]. Weekly work hours beyond 60 h/week were also explored as “extreme work hours,” as described in a prior study [44].

Usual work schedule was ascertained by respondents' self‐selection into one of four work schedule categories—regular daytime schedule, regular evening shift, regular night shift, and rotating shift [34]. We included this variable to better understand the prevalence of overnight work among those who do not self‐identify as regular night shift workers. “Years on the job” referred to time respondents have been employed in their main job or business. Respondents' work arrangement was described as a “regular, permanent employee,” “independent contractor, freelancer or consultant,” “working for a contractor,” or “other.” Employer type was categorized as “private company,” “government,” “self‐employed,” or “working without pay at a family‐owned business.” Industry and occupation were described in the NHIS using categories based on 4‐digit Census codes consistent with the 2012 North American Industry Classification System and the 2010 Standard Occupational Classification [33]. We further grouped industry categories into sectors identified in the National Occupational Research Agenda (NORA), an extensive NIOSH partnership program that includes stakeholders from industry, labor, academia, the practitioner community, and other governmental agencies to develop innovative research and workplace interventions [45, 46, 47].

### 2.3 Analysis

All statistical analyses were performed using SAS 9.4 survey procedures (SAS Institute, Cary, NC). To account for the complex survey design of the NHIS and create a representative sample of US workers, responses were weighted using the NHIS sample adult record weight “to adjust for design, ratio, nonresponse, and poststratification” [33]. As per NCHS guidelines, estimates and proportions were not reported for categories with 30 or fewer unweighted responses [48]. Confidence intervals were calculated using the Korn–Graubard method to account for the complex survey design of the NHIS [33, 49]. Prevalence rates were calculated from weighted estimates and represent the proportion of respondents who reported working an overnight shift in the past 30 days out of the total population of workers in the category of interest. Prevalence ratios represent the ratio of the category of interest to the reference category. Confidence intervals that include “1” indicate that the prevalence between groups is not significantly different. Global test of proportions, using the Rao–Scott modified *χ* ^2^ test, was conducted to examine the association of each variable with overnight work. The complex survey sampling design of the NHIS can introduce correlation among sample units, therefore using traditional *χ* ^2^ tests would be inappropriate. The Rao–Scott modification accounts for the complex design, thus resulting in more accurate *p* values [50, 51]. We defined statistically significant associations as *p* < 0.05.

This activity was reviewed by CDC, deemed research not involving human subjects, and was conducted consistently with applicable federal law and CDC policy.^1^

## 3 Results

**Figure 1:**
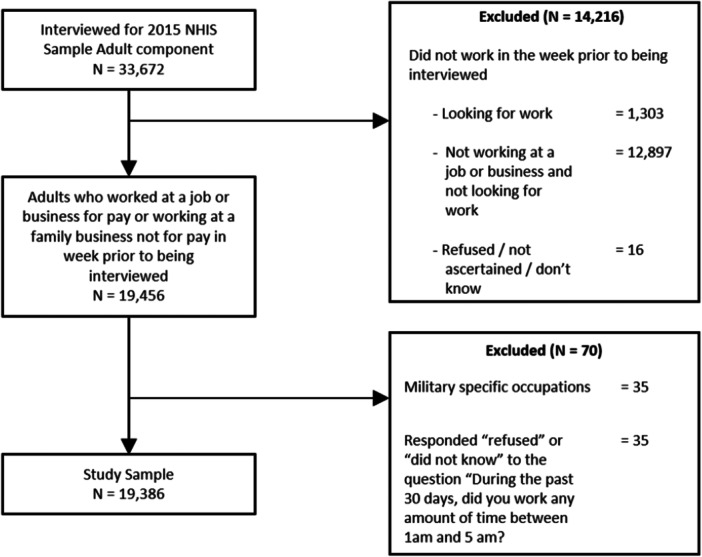
Sample size establishment from respondents to the 2015 National Health Interview Survey for estimates of prevalence of working any time between 1:00 a.m. and 5:00 a.m. in the past 30 days.

From the 33,672 adults who were interviewed for the 2015 NHIS, 19,456 reported working at a job or business for pay, with a job or business but not at work, or working at a family business not for pay in the week prior to being interviewed. We excluded those in military‐specific occupations (*N* = 35) because the NHIS does not assign a weight to estimate the national military population [33]. We further excluded those who did not provide a response to the overnight work question (*N* = 35). Our final sample population of workers consisted of 19,386 respondents (Figure 1). Among those, 2779 reported working any time between 1 a.m. and 5 a.m. in the 30 days prior to being interviewed. This represents over 21 million US adults and 14.2% of the total US worker population (Table 1).

**Table 1:** Sociodemographic characteristics for workers, 18 years and older, who reported working overnight (i.e., any amount of time between 1:00 a.m. and 5:00 a.m.) in the past 30 days.

	Unweighted sample population of respondents who worked overnight	Estimated national population who worked overnight	Weighted prevalence^b^ (95% CI)	Prevalence ratio (95% CI)	Modified Rao–Scott *ᴪ* ^2^ (*p*)
Total	2779	21,137,000	14.2 (13.5–15.0)	—	
Age (years)					**< 0.0001**
18–24	289	2,773,000	14.9 (12.6–17.3)	0.97 (0.81–1.16)	
25–34	720	5,029,000	15.6 (14.1–17.3)	1.02 (0.89–1.17)	
35–44	630	4,912,000	15.3 (13.8–16.9)	Reference	
45–54	617	4,725,000	14.2 (12.8–15.6)	0.92 (0.81–1.05)	
55–64	407	3,119,000	**12.6 (11.0–14.2)**	**0.82 (0.70–0.96)**	
≥ 65	116	579,000	**8.0 (6.2–10.1)**	**0.52 (0.40–0.67)**	
Sex					
Male	1720	13,942,000	**17.8 (16.6–19.0)**	**1.72 (1.56–1.90)**	**< 0.0001**
Female	1059	7,194,000	10.3 (9.5–11.1)	Reference	
Education					**< 0.0001**
Less than high school	215	1,612,000	13.1 (10.9–15.5)	1.13 (0.94–1.35)	
High school/GED	684	5,417,000	**16.7 (15.0–18.4)**	**1.43 (1.27–1.62)**	
Some college or associate degree	1004	7,556,000	**15.9 (14.6–17.3)**	**1.37 (1.21–1.55)**	
University degree	871	6,447,000	11.6 (10.6–12.7)	Reference	
Race and ethnicity					**< 0.0001**
Non‐Hispanic White	1786	14,015,000	14.5 (13.6–15.5)	Reference	
Non‐Hispanic Black	419	2,988,000	**17.2 (15.1–19.4)**	**1.19 (1.04–1.36)**	
Hispanic	392	2,793,000	**11.6 (10.1–13.1)**	**0.80 (0.69–0.92)**	
Non‐Hispanic Asian	140	1,084,000	12.1 (9.9–14.5)	0.83 (0.69–1.00)	
Non‐Hispanic American Indian/Alaska Native	a	—	—	—	
Non‐Hispanic Other Race	a	—	—	—	
Nativity					**< 0.0001**
US born	2356	18,011,000	15.0 (14.1–15.8)	Reference	
Non‐US born	423	3,125,000	**11.2 (9.9–12.6)**	**0.75 (0.66–0.85)**	
Marital status					0.43
Married/living with partner	1442	13,339,000	14.0 (13.0–14.9)	Reference	
Widowed/divorced/separated	541	2,852,000	15.0 (13.5–16.6)	1.07 (0.96–1.20)	
Never married	791	4,928,000	14.6 (13.3–16.1)	1.05 (0.94–1.17)	
Minor children in the family					0.55
No	1738	12,277,000	14.1 (13.2–15.0)	Reference	
Yes	1041	8,860,000	14.5 (13.4–15.6)	1.03 (0.94–1.13)	
Region of residence					0.12
Northeast	422	3,366,000	13.3 (11.8–14.9)	Reference	
Midwest	672	5,495,000	**15.8 (14.1–17.7)**	**1.19 (1.01– 1.40)**	
South	900	7,396,000	13.8 (12.6–15.1)	1.04 (0.90–1.21)	
West	785	4,879,000	14.0 (12.7–15.5)	1.06 (0.91–1.23)	

*Note:* Bold = significant differences.

Abbreviation: CI = confidence interval.

a Estimate not reported as per NCHS guidelines for < 30 responses in the sample population.

^b^ Weighted prevalence = estimated population for category of interest/total national population of workers for category of interest.

### 3.1 Sociodemographic Variables

Age, gender, education, race and ethnicity, and nativity were all significantly associated with overnight work (*p* < 0.0001, Table 1). A lower prevalence of overnight work was reported among workers aged 55–64 years (12.6%) and 65 years and over (8.0%), compared to those aged 35–44 years (15.3%). Significantly higher prevalence was found among men (17.8%) compared to women (10.3%). Workers with high school or equivalent education, or some college education, reported higher percentages of overnight work (16.7% and 15.9%, respectively) compared to those with university degrees (11.6%). Compared to non‐Hispanic White workers (14.5%), non‐Hispanic Black workers reported higher prevalence (17.2%), while lower rates were found among Hispanic workers (11.6%). Lower prevalence of overnight work also occurred among non‐US born workers (11.2%) compared to US born (15.0%). Higher prevalence was reported among workers living in the Midwest (15.8%) compared to the Northeast (13.3%). No significant differences were found for marital status, or presence of minor children in the family.

### 3.2 Health Status and Health Behaviors

Across health status and health behaviors, only sleep and smoking status were significantly associated with overnight work (both *p* < 0.0001, Table 2). Compared to workers who slept 7–9 h over the past 24 h (10.5%), significantly higher prevalence of overnight work occurred among those who slept < 7 h (21.4%) and more than 9 h (17.0%). Prevalence of current and former smoking was higher (19.3% and 15.7%, respectively) compared to never having smoked (12.7%). We also found that overnight workers reported a higher prevalence of fair or poor health (16.7%), compared to excellent or very good health. No significant differences in prevalence were found across categories of leisure‐time physical activity or current alcohol use.

**Table 2:** Health status and behaviors for workers, 18 years and older, who reported working overnight (i.e., any amount of time between 1:00 a.m. and 5:00 a.m.) in the past 30 days.

	Unweighted sample population of respondents who worked overnight	Estimated national population who worked overnight	Weighted prevalence^a^ (95% CI)	Prevalence ratio (95% CI)	Modified Rao–Scott *ᴪ* ^2^ (*p*)
Total	2779	21,137,000	14.2 (13.5–15.0)	—	
Self‐reported health					0.11
Excellent/Very good	1865	14,298,000	13.9 (13.0–14.7)	Reference	
Good	706	5,423,000	14.8 (13.4–16.3)	0.96 (0.78–1.18)	
Fair/poor	181	1,411,000	**16.7 (13.9–19.8)**	**1.21 (1.01–1.43)**	
Sleep					**< 0.0001**
< 7 h	1365	10,363,000	**21.4 (19.8–23.0)**	**2.04 (1.83–2.28)**	
7–9 h	1271	9,646,000	10.5 (9.6–11.3)	Reference	
> 9 h	49	426,000	**17.0 (11.6–23.7)**	**1.62 (1.14–2.31)**	
Leisure time physical activity					0.12
None	683	5,206,000	13.9 (12.5–15.3)	Reference	
Light/moderate	511	3,816,000	13.1 (11.6–14.6)	0.94 (0.81–1.09)	
Vigorous	1553	11,882,000	14.8 (13.8–15.8)	1.07 (0.95–1.20)	
Smoker					**< 0.0001**
Never	1656	12,455,000	12.7 (11.9–13.5)	Reference	
Former	567	4,406,000	**15.7 (14.2–17.3)**	**1.24 (1.11–1.39)**	
Current	549	4,196,585	**19.3 (17.2–21.5)**	**1.52 (1.34–1.72)**	
Current alcohol use					0.74
Infrequent	355	2,646,328	14.0 (12.2–15.9)	Reference	
Light	1001	7,921,095	14.7 (13.6–15.9)	1.05 (0.91–1.23)	
Moderate	548	4,190,738	15.3 (13.7–16.9)	1.09 (0.92–1.29)	
Heavy	162	1,128,086	14.3 (11.7–17.3)	1.03 (0.82–1.28)	

*Note:* Bold = significant differences.

Abbreviation: CI = confidence interval.

^a^ Weighted prevalence = estimated population for category of interest/total national population of workers for category of interest.

### 3.3 Occupational Characteristics

Not surprisingly, usual work schedule was associated with overnight work (*p* < 0.0001, Table 3). The highest prevalence of overnight work was reported by those who usually worked nights (77.6%), followed by rotating shift workers (30.4%) and evening shift workers (20.5%). However, almost 7% of regular daytime workers also worked sometime between 1 a.m. and 5 a.m. Working overnight was also associated with weekly hours worked (*p* < 0.0001, Table 3), with increased prevalence occurring with increasing hours of work. Lowest prevalence of overnight work was found among those working < 20 h/week (8.2%), with increasing percentages for 21–40 h/week (11.3%), 41–60 h/week (20.7%), and > 60 h/week (35.8%). Having more than one job was associated with overnight work (*p* < 0.0001), with higher prevalence among workers with multiple jobs, compared to those with one job (19.5% vs. 13.8%). Prevalence of overnight work was lower among government workers compared to those employed in private companies (12.2% vs. 14.6%). There were no significant findings for years on the job, work arrangement, or paid sick leave.

**Table 3:** Occupational characteristics for workers 18 years and older, who reported working overnight (i.e., any amount of time between 1:00 a.m. and 5:00 a.m.) in the past 30 days.

	Unweighted sample population of respondents who worked overnight	Estimated national population who worked overnight	Weighted prevalence^b^ (95% CI)	Prevalence ratio (95% CI)	Modified Rao–Scott *ᴪ* ^2^ (*p*)
Total	2779	21,137,000	14.2 (13.5–15.0)	—	
Weekly hours worked					**< 0.0001**
≤ 20	180	1,404,000	**8.2 (6.9–9.8)**	**0.73 (0.61–0.88)**	
21–40	1283	9,903,000	11.3 (10.4–12.1)	Reference	
41–60	1011	7,799,000	**20.7 (19.0–22.4)**	**1.83 (1.65–2.04)**	
> 60	305	2,031,000	**35.8 (31.3–40.4)**	**3.18 (2.74–3.68)**	
Usual work schedule					**< 0.0001**
Daytime	942	7,249,000	6.7 (6.1–7.3)	Reference	
Evening	193	1,580,000	**20.5 (16.7–24.8)**	**3.09 (2.49–3.82)**	
Night	557	4,406,000	**77.6 (72.8–82.0)**	**11.67 (10.54–12.92)**	
Rotating	1083	7,877,000	**30.4 (28.2–32.6)**	**4.56 (4.08–5.10)**	
Years on the job					0.34
0–5	1535	11,552,000	14.3 (13.3–15.3)	Reference	
6–10	488	3,677,000	13.9 (12.4–15.6)	0.98 (0.85–1.11)	
11–20	484	3,909,000	15.4 (13.8–17.2)	1.08 (0.95–1.23)	
20+	267	1,965,000	13.1 (11.1–15.2)	0.91 (0.77–1.08)	
Work arrangement					0.67
Regular, permanent employee	2285	17,428,000	14.2 (13.4–15.0)	Reference	
Independent contractor, freelance, or consultant	301	2,201,000	15.0 (12.9–17.3)	1.05 (0.91–1.23)	
Working for a contractor	80	648,000	15.6 (11.5–20.4)	1.10 (0.83–1.45)	
Other	112	855,000	12.8 (9.9–16.3)	0.90 (0.70–1.16)	
Employer type					**0.03**
Private company	2141	16,373,000	14.6 (13.7–15.5)	Reference	
Government (federal, state, or local)	364	2,692,000	**12.2 (10.6–14.0)**	**0.84 (0.71–0.98)**	
Self‐employed	255	1,982,000	15.2 (13.1–17.6)	1.04 (0.89–1.22)	
Family‐owned business without pay	a	—	—	—	
More than one job					**< 0.0001**
No	2447	18,724,000	13.8 (13.0–14.6)	Reference	
Yes	332	2,413,000	**19.5 (16.9–22.4)**	**1.42 (1.22–1.65)**	
Paid sick leave					0.74
No	1180	8,955,000	14.1 (13.1–15.3)	0.98 (0.89–1.08)	
Yes	1579	12,041,000	14.4 (13.4–15.4)	Reference	

*Note:* Bold = significant differences.

Abbreviation: CI = confidence interval.

^a^ Estimate not reported as per NCHS guidelines for < 30 responses in the sample population.

^b^ Weighted prevalence = estimated population for category of interest/total national population of workers for category of interest.

### 3.4 Industry and Occupation

The industries with the highest prevalence of overnight work were Transportation, Warehousing, and Utilities sector (29.3%), followed by Agriculture, Forestry, and Fishing (21.7%), Public Safety (19.9%), Manufacturing (19.3%), and Healthcare and Social Assistance (18.5%) (Table 4). The lowest prevalence of overnight work was reported in the Construction sector (8.4%), followed by Services (11.0%), and in some Service subsectors: Finance and Insurance (5.6%), Real Estate (6.3%), Education (4.9%), and Other Services (7.6%). These included Repair and maintenance, Personal services (e.g., barber shops, laundry, funeral homes), and Religious, grantmaking, civic, and labor services. However, other Service subsectors, such as Accommodation and Food (18.2%), reported a higher rate of overnight work compared to all US workers.

**Table 4:** Prevalence of overnight work (i.e., any amount of time between 1:00 a.m. and 5:00 a.m.) in the United States, among workers 18 years and older, in the past 30 days, by Industry and National Occupational Research Agenda (NORA) Sectors, 2015.

Industry sector	Unweighted sample population of respondents who worked overnight	Estimated national population who worked overnight	Weighted Prevalence^b^ (95% CI)	Prevalence Ratio^c^ (95% CI)
Total	2779	21,137,000	14.2 (13.5–15.0)	
Agriculture, Forestry, and Fishing	66	416,000	**21.7 (16.9–27.3)**	**1.54 (1.13–2.09)**
Construction	102	760,000	**8.4 (6.6–10.5)**	**0.58 (0.45–0.74)**
Healthcare and Social Assistance	499	3,586,000	**18.5 (16.7–20.4)**	**1.36 (1.22–1.51)**
Manufacturing	350	2,981,000	**19.3 (17.1–21.6)**	**1.41 (1.24–1.61)**
Mining	50	144,000	17.2 (11.4–24.4)	1.21 (0.80– 1.81)
Public Safety	186	1,449,000	**19.9 (17.4–22.6)**	**1.43 (1.20–1.70)**
Services	932	7,273,000	**11.0 (10.1–11.9)**	**0.65 (0.59–0.71)**
Information	67	567,000	17.2 (13.7–21.2)	1.21 (0.91–1.62)
Finance and Insurance	48	398,000	**5.6 (4.1–7.4)**	**0.38 (0.26–0.54)**
Real Estate and Rental and Leasing	33	194,000	**6.3 (4.1–9.1)**	**0.44 (0.29–0.65)**
Professional, Scientific and Technical	183	1,488,000	13.1 (10.9–15.5)	0.91 (0.76–1.10)
Management of Companies and Enterprises	a	—	—	—
Administrative and Support and Waste Management and Remediation	140	1,074,000	15.9 (13.1–19.0)	1.12 (0.90–1.39)
Education	100	676,000	**4.9 (3.8–6.1)**	**0.32 (0.25–0.41)**
Arts, Entertainment and Recreation	59	425,000	15.3 (11.8–19.4)	1.08 (0.78–1.48)
Accommodation and Food	222	1,873,000	**18.2 (15.7–20.9)**	**1.31 (1.10–1.55)**
Other services	80	577,000	**7.6 (5.7–10.0)**	**0.52 (0.38–0.72)**
Transportation, Warehousing and Utilities	266	1,937,000	**29.3 (25.9–32.9)**	**2.16 (1.88–2.49)**
Utilities	45	267,000	**24.6 (18.2–32.0)**	**1.74 (1.17–2.58)**
Transportation and Warehousing	221	1,670,000	**30.2 (26.7–33.9)**	**2.22 (1.90–2.58)**
Wholesale and Retail Trade	301	2,400,000	12.4 (10.6–14.4)	0.86 (0.73–1.01)
Wholesale Trade	54	508,000	12.5 (8.9–16.9)	0.87 (0.61–1.25)
Retail Trade	247	1,892,000	12.4 (10.6–14.4)	0.86 (0.73–1.01)

*Note:* Main headings reflect National Occupational Research Agenda (NORA) sector categories (https://www.cdc.gov/nora/default.html). Subheadings represent industries within each NORA sector. Results for the Oil and Gas Extraction sector have not been reported because the microdata is not available in the public use data set.

b No responses reported.

^c^ Weighted prevalence = estimated population for category of interest/total national population of workers for category of interest.

^d^ Prevalence Ratio is based on comparison to all other US workers, not in the Sector.

Occupations reporting higher rates of working between 1:00 a.m. and 5:00 a.m., in comparison to all US workers, included Protective Services (47.4%), Transportation and Material Moving (28.8%), Healthcare Practitioners and Technical (26.3%), Production (21.3%), Installation, Maintenance and Repair (20.5%), and Healthcare Support (18.6%) (Table 5). Those with significantly lower rates include Sales and related (10.1%), Construction and Extraction (9.8%), Building, Grounds Cleaning and Maintenance (9.3%), Office and Administrative Support (8.2%), Business and Financial Operations (6.1%), and Education (4.2%).

**Table 5:** Occupations of workers 18 years and older who reported working between 1:00 a.m. and 5:00 a.m. in the 30 days before participating in the National Health Interview Survey, in the United States, 2015.

	Unweighted sample population of respondents who worked overnight	Estimated national population who worked overnight	Weighted prevalence^b^ (95% CI)	Prevalence ratio^c^ (95% CI)
Total	2779	21,137,000	14.2 (13.5–15.0)	
Architecture and engineering	34	366,000	10.8 (7.8–14.4)	0.75 (0.51–1.11)
Arts, design, entertainment, sports and media	67	469,000	15.1 (11.8–18.9)	1.06 (0.79–1.42)
Building and grounds cleaning and maintenance	69	511,000	**9.3 (6.9–12.1)**	**0.64 (0.46–0.91)**
Business and financial operations	67	482,000	**6.1 (4.7–7.8)**	**0.41 (0.31–0.55)**
Community and social services	46	317,000	10.7 (7.0–15.6)	0.75 (0.49–1.14)
Computer and mathematical	96	776,000	14.8 (11.9–18.1)	1.04 (0.82–1.32)
Construction and extraction	108	663,000	**9.8 (7.6–12.3)**	**0.68 (0.52–0.88)**
Education, training, and library	64	398,000	**4.2 (2.9–5.7)**	**0.28 (0.20–0.39)**
Farming, fishing, and forestry	41	215,000	20.2 (14.6–26.9)	1.42 (0.95–2.14)
Food preparation and serving related	139	1,109,000	14.8 (12.3–17.5)	1.04 (0.82–1.31)
Healthcare practitioners and technical	293	2,269,000	**26.3 (23.3–29.5)**	**1.95 (1.71–2.22)**
Healthcare support	98	630,000	**18.6 (15.3–22.3)**	**1.31 (1.01–1.71)**
Installation, maintenance, and repair	113	1,013,000	**20.5 (17.1–24.2)**	**1.46 (1.17–1.82)**
Legal	a	—	—	—
Life, physical, and social science	a	—	—	—
Management	274	2,099,000	13.7 (12.0–15.6)	0.96 (0.83–1.11)
Office and administrative support	208	1,430,000	**8.2 (6.9–9.6)**	**0.54 (0.46–0.64)**
Personal care and service	97	585,000	12.0 (9.5–14.9)	0.84 (0.62–1.13)
Production	248	1,927,000	**21.3 (18.6–24.2)**	**1.54 (1.33–1.79)**
Protective service	156	1,347,000	**47.4 (41.5–53.4)**	**3.49 (2.92–4.16)**
Sales and related	180	1,520,000	**10.1 (8.5–12.0)**	**0.69 (0.57–0.83)**
Transportation and material moving	305	2,391,000	**28.8 (25.7–32.0)**	**2.15 (1.88–2.46)**

*Note:* Bold = significant differences.

Abbreviation: CI = confidence interval.

^a^ Estimate not reported as per NCHS guidelines for < 30 responses in the sample population.

^b^ Weighted prevalence = estimated national population for category of interest/estimated total national population of workers

^c^ Prevalence ratio is based on comparison to all other US workers, not in the Sector.

## 4 Discussion

This study used data from the 2015 NHIS and found that 14% of US workers experienced work occurring between 1:00 a.m. and 5:00 a.m. Our definition of overnight work reflects the “window of circadian low,” when consistent wakefulness may have the most deleterious health and safety effects [29, 30, 31, 32, 52, 53]. A precise definition of overnight work exposure agreed upon by subject matter experts, government agencies, and professional organizations may provide a better understanding of determinants of occupational health and safety risks [27].

Our prevalence estimate is slightly lower than estimates from a study using pooled NHANES data from 2005 to 2010. While we estimated 14% of workers experienced overnight work, the NHANES study found that 17% of US workers reported usually working between 5:00 p.m. and 8:00 a.m. [54]. This minor difference could be attributed to differences in night shift definitions. However, our results were almost five times higher than estimates using the 2004 CPS which reported 3.1% of workers were regularly employed in night shifts (defined as usual hours of work between 9 p.m. and 8 a.m.) [21]. Differences in prevalence across studies suggest there has been a substantial increase in the number of overnight workers from 2004 to 2015. Given that overnight work has been associated with adverse safety and health outcomes, additional policies and programs may be needed to protect this growing population of workers.

Across sociodemographic characteristics, a lower prevalence of overnight work was reported among older age groups. This finding is similar to prior studies and may reflect a higher seniority in their organization and opportunities for “more desirable” circadian‐ and socially‐aligned jobs (e.g., management) and work schedules (e.g., regular daytime shifts) [18, 21, 55]. Our findings of higher prevalence of overnight work among men are consistent with findings reported in prior studies using data from the 1991, 1997, and 2004 CPS [18, 19, 20, 21]. However, our estimates for both men and women employed in overnight work (17.9% and 10.4%) were substantially higher compared to estimates from the 2004 CPS (3.5% for men and 2.6% for women). The discrepancy in findings may reflect differences in the definition of overnight work between the data sets, but more likely due to workforce characteristics and types of jobs available between the years the CPS and NHIS were administered (i.e., 1991, 1997, and 2004, vs. 2015). Gender differences in overnight work have been mainly attributed to more men employed in industries and occupations with nonstandard schedules [18]. It has also been suggested that women take family responsibilities into account when making labor force decisions, such as work schedules [56, 57]. Presser (1991) found that women who were married or had preschool‐aged children had a lower likelihood of working at night [18]. To investigate possible reasons for gender differences in overnight work, we completed additional analyses and found that gender was significantly associated with industry and occupation (*p* < 0.0001, results not shown). For example, in the Transportation sector, where nonstandard schedules are common, we found that workers were predominantly men (79%), and men were almost twice as likely to be working overnights than women (OR 1.94, 95% CI: 1.43–2.64, results not shown). We also examined gender differences in security work which also requires around‐the‐clock service. Results showed that more men were employed in protective services than women (82% vs 18% respectively, *p* < 0.0001, results not shown), and overnight workers were twice as likely to be men than women (OR 2.26, 95% CI: 1.62–3.16, results not shown).

We found a higher prevalence of overnight work among some populations with increased risk for adverse occupational safety and health outcomes. Similar to prior studies, we report higher rates of overnight work among non‐Hispanic Black workers, which may reflect the types of occupations in which these populations are most likely to be employed [19, 20, 21]. In 2018, the Bureau of Labor Statistics reported that Black workers represented more than one‐quarter of those employed as nurses and health aides which may require work at all hours [58]. In a post hoc analysis, we found that race and ethnicity were significantly associated with industry and occupation (*p* < 0.0001, results not shown). Specifically, we found that compared to other races and ethnicities, a larger proportion of workers in Healthcare Support occupations were non‐Hispanic Black workers (16%, *p* < 0.0001, results not shown), compared to all other races and ethnicities. Furthermore, overnight workers in this sector were more than twice as likely to be non‐Hispanic Black (OR 2.24, CI: 1.58–3.17).

Our findings of a significant association between education level and overnight work are supported by Daghlas and colleagues, who reported the likelihood of frequent night shift work almost tripled with 3.6 fewer years of education, and may be mediated by occupational attainment [59]. We estimated a lower prevalence of overnight work among non‐US born workers, compared to US‐born workers. However, the underlying reasons are unclear. Recent BLS data also reported that in comparison to US‐born workers, there is slightly higher prevalence of non‐US born workers employed in “Farming, fishing, and forestry” occupations, which require overnight and early morning work (0.5% vs. 1.3%, respectively) [60].

We found that a larger proportion of overnight workers experience short sleep (< 7 h) and long sleep (> 9 h), and is similar to that in prior literature [6, 7, 14, 61, 62]. Work at night can result in significant shifts in sleep timing with negative effects on sleep quality and duration [8, 12]. Short sleep is frequently reported as a consequence of night shifts and has been attributed to circadian disruption and poorer daytime sleep [17, 63]. Longer sleep durations may reflect the need for longer recovery from night shifts among workers who have opportunities for sufficient recovery [64]. However, the NHIS question used to ascertain sleep duration asked about total sleep over a 24‐h period. Therefore, we were unable to determine if sleep was obtained in one episode, or as multiple, short fragments across different parts of the day, and whether participants were reporting sleep on a workday, a non‐workday, or an average of both. A recent study of sleep patterns among workers following a night shift reported that more than half of the participants engaged in biphasic or polyphasic sleep episodes [65]. It is unclear if our study population obtained sleep during work hours (when possible) or after work hours. While napping during night shifts is associated with lower levels of sleepiness at work, maintenance of alertness and performance, and may compensate for shortened sleep‐recovery periods while not at work [7, 63, 66, 67], obtaining multiepisodic sleep is not recommended for optimal health. Following an extensive review of the literature, the National Sleep Foundation issued a consensus statement which advised against fragmenting sleep into multiple episodes during the 24‐h day because “polyphasic sleep schedules, and the sleep deficiency inherent in those schedules, are associated with a variety of adverse physical health, mental health, and performance outcomes” [68].

Our findings confirm prior literature which reported poorer health and health behaviors among night shift workers, compared to regular daytime workers [1, 2, 7, 62]. Similarly, studies have demonstrated that smoking is a mediator between shift work and poor health [69]. Our finding that smoking behavior is associated with overnight work supports findings in prior studies and reviews [62, 70, 71]. Compared to daytime workers, those working nonstandard shifts such as nights, have been reported to be more likely to start smoking and remain smokers, although the underlying causes are not clear [70]. It is hypothesized that the use of smoking or other nicotine products to counteract sleepiness may help to adjust the biological clock to changing work and sleep schedules [62, 70, 72]. However, smoking has also been found to adversely impact sleep quality [73].

Work schedule, not surprisingly, was significantly associated with overnight work. However, among respondents identifying as regular night shift workers, ~20% did not report working overnight in the past 30 days. It may be that those identifying as “regular night shift workers” may not have worked between 1:00 a.m. and 5:00 a.m. Similarly, some “regular daytime workers” reported working between 1:00 a.m. and 5:00 a.m. may include those who start work in the early morning hours. This provides an example of how self‐reporting with broad categories may result in exposure misclassification, which can further bias study findings [28]. Additionally, our finding that over 15 million workers in rotating shift and regular day schedules also experience overnight work highlights that many workers may face the same, or even greater, adverse health and safety risks as those who regularly work nights. Therefore, efforts to reduce the risks associated with overnight or early morning shifts may be beneficial for all workers, regardless of their regular work schedule.

Our finding that overnight work is associated with increasing work hours raises concerns about their combined negative effects [9, 10, 11, 17]. Prior literature has found that for many workers, the primary reason for working overnight and longer hours is due to “the nature of the job” [19, 21, 74]. Secondary motivators include increased pay and personal preference [19, 21, 74]. While we found that almost 7% of overnight workers also worked more than 40 h/week, this is more than double the prevalence reported by Presser in 1991 (2.9%). This may suggest a rise in nonstandard work hours and extended shifts. It may also have resulted from the increased prevalence of multiple jobs, as some workers may take on overnight work in addition to daytime work for extra income [75]. Additional jobs can result in more time spent working various shifts and commuting between jobs, less time for sleep and recovery, with increased risk for occupational fatigue and injury [76]. Concerningly, while we found that almost 1 in 5 workers are employed in multiple jobs, an increase in multiple job holdings has been predicted as the economy “moves toward short‐term labor models and online contract platforms grow across industries” [77, 78].

Overnight work has been strongly attributed to industry and occupational requirements. Presser found that industry and occupation are important determinants of nonstandard work schedules, more so than socioeconomic factors such as sex, marital status, presence of children, ethnicity/race, and education [18, 19]. Our findings of higher prevalence of overnight work among industries and occupations which require work at all hours of the day and night is similar to prior studies which used data from the 1991 and 1997 CPS. However, while we found the greatest prevalence among the Transportation, Warehousing, and Utilities sector, prior studies reported a higher proportion of overnight shifts among some Services (i.e., Protective Services) [18, 20]. Differences between study findings may have occurred due to differences in the survey questions, or because of changing workforce demands (e.g., types of jobs and tasks within jobs) and rise in the “just‐in‐time” workforce with consequences for work scheduling [35].

## 5 Strengths and Limitations

This study uses a large, nationally representative survey of the US population which allowed for reliable estimates of prevalence of overnight work across most sociodemographic, health and occupational characteristics. Our definition of overnight work was more precise than in prior studies, centering around the window of circadian low, and may have reduced the potential for exposure misclassification. However, responses were self‐reported for occurrences in the 30 days prior to being interviewed and may have been limited by recall bias. As such, this may have resulted in an underestimation of the true prevalence of overnight work. There may be concerns that the data for this study were collected in 2015 and may be dated. However, the definition of overnight work (i.e., any work between 1:00 a.m. and 5:00 a.m.), based on biological theories of circadian rhythms, has not been repeated in subsequent years of the NHIS [27, 29, 30, 31, 32]. As such, this study provides the most recent update on overnight work in the USA.

## 6 Conclusions

Our findings suggest that there has been a substantial increase in overnight work in 2015, compared to earlier studies. However, it is unclear which factors may be the most prominent drivers of overnight work between these periods. It would be beneficial to regularly collect information on nonstandard work schedules, such as overnight work, in the NHIS to better identify changing trends, determinants, and risk associated with work hours. We encourage more current surveillance of nonstandard work schedules as technology and global demands change the way we work. It has been suggested that new technologies enable constant connectivity across time zones, blurring the boundaries between work and leisure, and often leading to longer or fragmented work days which may include overnight hours [79]. It is also believed that unpredictable workloads and demand for around‐the‐clock accessibility have led to the development of flexible workforces and a shift toward shorter‐term, alternative work arrangements [80]. These precarious and nonstandard schedules, in turn, can have adverse effects on work–life balance and worker well‐being [81, 82]. To gain empirical evidence of the trends associated with the changing nature of work, it is imperative to include the same detailed questions about work scheduling and practices at regular intervals in national surveys. Studying trends over time can enable us to better understand the future demands on our workforce and what occupational safety and health policies and programs may need to be revised or developed.

## Author Contributions

Imelda Wong led all aspects of this study including conceptualizing the study idea, developing the study methodology, leading the statistical analyses, drafting the original manuscript, and revising all versions. Toni Alterman provided senior authorship guidance in using NHIS data, co‐conceptualized the study idea, co‐developed the study methodology, suggested salient socioeconomic, health, and occupational variables to examine for overnight workers, and reviewed all versions of this manuscript. Beverly Hittle co‐conceptualized the study idea, assisted with identifying variables for analyses, and reviewed all versions of the manuscript. Raquel Velazquez‐Kronen assisted with refining the methodology, assisted with identifying variables for analyses and reviewed all versions of the manuscript. I‐Chen Chen co‐led the analyses, conducted all statistical calculations, and reviewed all versions of the manuscript.

## Disclosure

This work was prepared while Dr. Imelda Wong, Dr. I‐Chen Chen, and Dr. Velazquez‐Kronen were employed at the National Institute for Occupational Safety and Health, Centers for Disease Control and Prevention. The findings and conclusions in this paper are those of the authors and do not necessarily represent the views of the National Institute for Occupational Safety and Health, National Center for Health Statistics, Centers for Disease Control and Prevention, State of Hawaii, Department of Human Services, or the Office of the Provincial Health Officer, Ministry of Health, British Columbia Government.

## Ethics Statement

This paper used free, publicly available deidentified data and therefore did not require ethics approval. The NHIS is approved by the Research Ethics Review Board of the National Center for Health Statistics, Centers for Disease Control and Prevention, and the US Office of Management and Budget.

## Conflicts of Interest

The authors declare no conflicts of interest.

## Supporting information

Supplement A variables questions responses 24 20 08.

## Data Availability

The data that support the findings of this study are openly available in NHIS Data Release at https://archive.cdc.gov/www_cdc_gov/nchs/nhis/nhis_2015_data_release.htm.

## Endnotes

1 See e.g., C.F.R. part 46; 21 C.F.R. part 56; 42 U.S.C. §241(d), 5 U.S.C. §552a, 44 U.S.C. §3501 et seq.
