# Supplementary material for: Prevalence of Overnight Work (1 a.m. to 5 a.m.) Among United States Workers
Source: Am J Ind Med. 2025 Oct 7;68(12):1088–104. doi: 10.1002/ajim.70027 (PMC12606400; doi:10.1002/ajim.70027)
Supplement: Supplementary file 1 — Supplement A variables questions responses 24 20 08. [file AJIM-68-1088-s001.docx]

**Supplement A** – Variables and questions from 2015 National Health Interview Survey used to estimate sociodemographic, health status and health behaviors and occupational characteristics of respondents who worked any amount of time between 1 am and 5 am in the past 30 days prior to being interviewed.

| **Variable** | **NHIS variable name** | **Question** | **NHIS Responses and category coding** |
| --- | --- | --- | --- |
| Employment Status Last Week | doinglwa | No question text. NHIS recoded variable “Corrected Employment Status Last Week” | **1** Working for pay at a job or business  **2** With a job or business but not at work  **3** Looking for work  **4** Working, but not for pay, at a family-owned job or business  **5** Not working at a job or business and not looking for work  **7** Refused  **8** Not ascertained  **9** Don't know |
| Overnight shift | nightwk | During the past 30 days, did you work any amount of time between 1:00 am and 5:00 am? | **1** Yes  **2** No  **7** Refused  **9** Don't know |
| Age | age_p | What is your age? | Continuous variable from 1-84. Over 85 is coded as a separate category. |
| Sex | sex | Are you male or female? | **1** Male  **2** Female |
| Region | region | No associated question. Information gathered from household component. |  |
| Race and Ethnicity | hiscodi3, mracrpi2 | Do you consider yourself to be Hispanic or Latino?  What race or races do you consider yourself to be? | NHIS recoded responses by combining hiscodi3 and mracrpi2.  **1** Non-Hispanic White  **2** Non-Hispanic Black  **3** Hispanic  **4** Non-Hispanic Asian/Native Hawaiian or Other Pacific Islander  **5** Non-Hispanic American Indian/Alaska Native  **6** Non-Hispanic Other Race |
| Nativity | plborn | Were you born in the United States? | **1** Yes  **2** No  **7** Refused  **8** Not ascertained  **9** Don't know |
| Marital status | r_maritl | Are you now married, widowed, divorced, separated, never married, or living with a partner? | **1** Married - spouse in household  **2** Married - spouse not in household  **3** Married -spouse in household unknown  **4** Widowed  **5** Divorced  **6** Separated  **7** Never married  **8** Living with partner  **9** Unknown marital status |
| Parent to 1+ minor child(ren) in the family | par_stat | No associated question. Information derived from household composition. | **1** Yes, the Sample Adult is a parent of a child residing in the family  **2** There are minor children residing in the family but the Sample Adult is not their parent  **3** There are no minor children residing in the family  **9** Unknown |
| Educational attainment | educ_1 | What is the HIGHEST level of school you have completed or the highest degree you have received? | **00** Never attended/kindergarten only  **1** 1st grade  **2** 2nd grade  **3** 3rd grade  **4** 4th grade  **5** 5th grade  **6** 6th grade  **7** 7th grade  **8** 8th grade  **9** 9th grade  **10** 10th grade  **11** 11th grade  **12** 12th grade, no diploma  **13** GED or equivalent  **14** High School Graduate  **15** Some college, no degree  **16** Associate degree: occupational, technical, or vocational program  **17** Associate degree: academic program  **18** Bachelor's degree (Example: BA, AB, BS, BBA)  **19** Master's degree (Example: MA, MS, MEng, MEd, MBA)  **20** Professional School degree (Example: MD, DDS, DVM, JD)  **21** Doctoral degree (Example: PhD, EdD)  **96** Child under 5 years old  **97** Refused  **99** Don't know” |
| Self-reported health status | phstat | Would you say your health in general is excellent, very good, good, fair, or poor? | **1** Excellent  **2** Very good  **3** Good  **4** Fair  **5** Poor  **7** Refused  **9** Don't know |
| Physical activity | vigfreqw  modfreqw | How often do you do VIGOROUS leisure-time physical activities for AT LEAST 10 MINUTES that cause HEAVY sweating or LARGE increases in breathing or heart rate?  How often do you do LIGHT OR MODERATE LEISURE-TIME physical activities for AT LEAST 10 MINUTES that cause ONLY LIGHT sweating or a SLIGHT to MODERATE increase in breathing or heart rate? | **0** Less than once per week  **1-28** times per week  **95** Never  **96** Unable to do vigorous activity  **97** Refused  **98** Not ascertained  **99** Don't know |
| Sleep | acisleep | On average, how many hours of sleep do you get in a 24-hour period? | **1-24** hours  **97** Refused  **98** Not ascertained  **99** Don't know |
| Smoking | smkstat2 | NHIS recoded variable: (Cigarette smoking status) classifies sample adults in terms of their lifetime and current cigarette smoking status. Categories include: current every day; current some days; former; never; {ever} smoker, current status unknown; and unknown if ever smoked {unknown lifetime status}. As noted earlier, current smokers are defined as persons who have ever smoked 100 cigarettes and who currently smoke every day or some days. Never smokers are defined as persons who never smoked any cigarettes or who have ever smoked less than 100 cigarettes. | **1** Current every day smoker  **2** Current some day smoker  **3** Former smoker  **4** Never smoker  **5** Smoker, current status unknown  **9** Unknown if ever smoked |
| Alcohol use | alcstat | NHIS recoded variable:  (Alcohol drinking status) classifies sample adults in terms of their lifetime and current alcohol drinking status. | **1** Lifetime abstainer  **2** Former infrequent  **3** Former regular  **4** Former, unknown  **5** Current infrequent  **6** Current light  **7** Current moderate  **8** Current heavier  **9** Current drinker, frequency/level unknown  **10** Drinking status unknown |
| Weekly work hours | wrkhrs2 | How many hours did you work LAST WEEK at ALL jobs or businesses/did ALIAS work LAST WEEK at ALL jobs or businesses/do you USUALLY work at ALL jobs or businesses? | **1-94** hours  **95+** hours  **97** Refused  **98** Not ascertained  **99** Don't know |
| Work schedule | wrksched | Which of the following best describes the hours you usually work? | **1** A regular daytime schedule  **2** A regular evening shift  **3** A regular night shift  **4** A rotating shift  **7** Refused  **9** Don't know |
| Years on the job | yrswrkpa | About how long have you worked at this MAIN job or business? | **0** Less than 1 year  **1-34** years  **35** or more years  **97** Refused  **98** Not ascertained  **99** Don't know |
| Work arrangement | wrkarr_p | Which of the following best describes your work arrangement? | **1** You work as an independent contractor, independent consultant, or freelance worker  **2** You are paid by a temporary agency  **3** You work for a contractor who provides workers and services to others under contract  **4** You are a regular, permanent employee (standard work arrangement)  **5** Some other work arrangement  **7** Refused  **9** Don't know |
| Employer type | wrkcata | Which of these best describes your current job or work situation? | **1** Employee of a PRIVATE company for wages  **2** A FEDERAL government employee  **3** A STATE government employee  **4** A LOCAL government employee  **5** Self-employed in OWN business, professional practice or farm  **6** Working WITHOUT PAY in a family-owned business or farm  **7** Refused  **8** Not ascertained  **9** Don't know |
| More than one job | onejob | Do you have more than one job or business? | **1** Yes  **2** No  **7** Refused  **8** Not ascertained  **9** Don't know |
| Paid sick leave | pdsicka | Do you have paid sick leave on this MAIN job or business? | **1** Yes  **2** No  **7** Refused  **8** Not ascertained  **9** Don't know |
| Industry | indstrn2 | During the course of the interview, verbatim responses were obtained from each eligible sample adult regarding his/her industry and occupation. This information was subsequently reviewed by U.S. Census Bureau coding specialists, who assigned appropriate industry and occupation codes. These codes, developed by U.S. Census Bureau staff for use in non-economic Federal surveys, are 4-digit Census codes for industry and occupation consistent with the 2012 North American Industry Classification System (NAICS) and 2010 Standard Occupational Classification (SOC). However, these are not actual NAICS and SOC codes. | **1** Agriculture, Forestry, Fishing, and Hunting Industries  **2** Mining Industries  **3** Utilities Industries  **4** Construction Industries  **5** Manufacturing Industries  **6** Wholesale Trade Industries  **7** Retail Trade Industries  **8** Transportation and Warehousing Industries  **9** Information Industries  **10** Finance and Insurance Industries  **11** Real Estate and Rental and Leasing Industries  **12** Professional, Scientific, and Technical Services Industries  **13** Management of Companies and Enterprises Industries  **14** Administrative and Support and Waste Management and Remediation Services  **15** Education Services Industries 16 Health Care and Social Assistance Industries  **17** Arts, Entertainment, and Recreation Industries  **18** Accommodation and Food Services Industries  **19** Other Services (except Public Administration) Industries  **20** Public Administration Industries  **97** Refused, classified  **98** Not ascertained  **99** Don't know |
| Occupation | occupn2 |  | **1** Management Occupations  **2** Business and Financial Operations Occupations  **3** Computer and Mathematical Occupations  **4** Architecture and Engineering Occupations  **5** Life, Physical, and Social Science Occupations  **6** Community and Social Services Occupations  **7** Legal Occupations  **8** Education, Training, and Library Occupations  **9** Arts, Design, Entertainment, Sports and Media Occupations  **10** Healthcare Practitioners and Technical Occupations  **11** Healthcare Support Occupations  **12** Protective Service Occupations  **13** Food Preparation and Serving Related Occupations  **14** Building and Grounds Cleaning and Maintenance Occupations  **15** Personal Care and Service Occupations  **16** Sales and Related Occupations  **17** Office and Administrative Support Occupations  **18** Farming, Fishing, and Forestry Occupations  **19** Construction and Extraction Occupations  **20** Installation, Maintenance, and Repair Occupations  **21** Production Occupations  **22** Transportation and Material Moving Occupations  **97** Refused, classified  **98** Not ascertained  **99** Don’t know |

Sample adult layout file

<https://ftp.cdc.gov/pub/Health_Statistics/NCHS/Dataset_Documentation/NHIS/2015/samadult_layout.pdf>
